# Acute and critically ill peripartum cardiomyopathy and 'bridge to' therapeutic options: a single center experience with intra-aortic balloon pump, extra corporeal membrane oxygenation and continuous-flow left ventricular assist devices

**DOI:** 10.1186/cc10098

**Published:** 2011-03-10

**Authors:** Sofie Gevaert, Yves Van Belleghem, Stefaan Bouchez, Ingrid Herck, Filip De Somer, Yasmina De Block, Fiona Tromp, Els Vandecasteele, Floor Martens, Michel De Pauw

**Affiliations:** 1 Department of Cardiology, Heart Center, Ghent University Hospital, De Pintelaan 185, 9000 Ghent, Belgium; 2 Department of Cardiac Surgery, Heart Center, Ghent University Hospital, De Pintelaan 185, 9000 Ghent, Belgium; 3 Department of Anesthesiology, Heart Center, Ghent University Hospital, De Pintelaan 185, 9000 Ghent, Belgium; 4 Department of Intensive Care, Heart Center, Ghent University Hospital, De Pintelaan 185, 9000 Ghent, Belgium

## Abstract

**Introduction:**

Peripartum cardiomyopathy (PPCM) patients refractory to medical therapy and intra-aortic balloon pump (IABP) counterpulsation or in whom weaning from these therapies is impossible, are candidates for a left ventricular assist device (LVAD) as a bridge to recovery or transplant. Continuous-flow LVADs are smaller, have a better long-term durability and are associated with better outcomes. Extra corporeal membrane oxygenation (ECMO) can be used as a temporary support in patients with refractory cardiogenic shock. The aim of this study was to evaluate the efficacy and safety of mechanical support in acute and critically ill PPCM patients.

**Methods:**

This was a retrospective search of the patient database of the Ghent University hospital (2000 to 2010).

**Results:**

Six PPCM-patients were treated with mechanical support. Three patients presented in the postpartum period and three patients at the end of pregnancy. All were treated with IABP, the duration of IABP support ranged from 1 to 13 days. An ECMO was inserted in one patient who presented with cardiogenic shock, multiple organ dysfunction syndrome and a stillborn baby. Two patients showed partial recovery and could be weaned off the IABP. Four patients were implanted with a continuous-flow LVAD (HeartMate II^®^, Thoratec Inc.), including the ECMO-patient. Three LVAD patients were successfully transplanted 78, 126 and 360 days after LVAD implant; one patient is still on the transplant waiting list. We observed one peripheral thrombotic complication due to IABP and five early bleeding complications in three LVAD patients. One patient died suddenly two years after transplantation.

**Conclusions:**

In PPCM with refractory heart failure IABP was safe and efficient as a bridge to recovery or as a bridge to LVAD. ECMO provided temporary support as a bridge to LVAD, while the newer continuous-flow LVADs offered a safe bridge to transplant.

## Introduction

Peripartum cardiomyopathy (PPCM) is a rare disease that affects women in the last month of their pregnancy or in the early puerpium (up to five months after delivery); it is characterized by left ventricular systolic dysfunction and symptoms of heart failure without any identifiable cause of heart failure. The incidence varies from 1:15,000 to 1:1,300 deliveries in some African countries and 1:299 in Haiti and is thought to be lower in Europe [1,2]. The historically bad prognosis with mortality rates ranging from 4 to 80% has improved because of advances in heart failure treatment [3].

Although already described in the 19^th ^century the condition was only defined as Peripartum Cardiomyopathy in 1971 by Demakis *et al*., who also proposed diagnostic criteria that later were confirmed during the 'Peripartum Cardiomyopathy: National Heart Lung and Blood Institute and Office of Rare Disease Workshop' in 2000 [4]. Several etiologies have been proposed comprising myocarditis, auto-immune mechanisms and pregnancy associated hormonal changes [5-7]. Recent data support the hypothesis that PPCM may develop as a result of complex interactions of pregnancy-associated factors against a susceptible genetic background [8,9]. The oxidative stress-cathepsin D-16 kDa prolactin hypothesis has been raised as a possible common pathway on which different etiologies that induce PPCM may merge. While newer therapies such as bromocriptine appear promising and will be tested in larger trials one must also concentrate on an optimal treatment strategy for the acute and critically ill PPCM patients, allowing to increase survival in this young patient population [10].

Heart transplantation is an accepted treatment option for patients with refractory heart failure due to PPCM, although a higher incidence of rejection has been reported in parous women, particularly in the first six months after transplantation [11,12]. Moreover, heart transplantation is limited by a lack of suitable donors. On the other hand there is a reasonable possibility of partial or complete recovery of left ventricular function, during the first year. The main predictors for recovery are an initial left ventricular end-diastolic dimension <56 mm and an ejection fraction >45% at two months [3]. As a consequence there is a need for appropriate temporary short- and long-term artificial support for the acute and critically ill patients. There are only a few reports on mechanical support devices as a bridge to recovery or transplantation in this setting. Data on the use of intra aortic balloon pump (IABP) and extra corporeal membrane oxygenation (ECMO) in PPCM are scarce [13-16]. There are a few reports on the use of pulsatile assist devices in this setting, most of them as a bridge to transplant and in a minority of cases as bridge to recovery [17-24].

Continuous-flow LVADs are a newer type of assist devices that have advantages over the older pulsatile devices: they are smaller, have a better long-term durability and their use is associated with improved survival and functional capacity [25,26]. There are no published series on the use of a continuous-flow device in patients with PPCM.

## Materials and methods

A retrospective 10-year study (2000 to 2010) was conducted of our patient database (Department of Cardiology, Ghent University Hospital, Belgium) for patients with a need for mechanical support in the acute phase of PPCM. Mechanical support was defined as IABP, ECMO or LVAD. We received local Ethical Committee approval and informed consent from the patients or their relatives.

Diagnosis of PPCM was based upon development of symptoms of heart failure due to systolic dysfunction in the last month of pregnancy or within five months after delivery without any identifiable cause of heart failure or recognizable heart disease prior to the last month of pregnancy. Patients with hypertensive heart failure in the peripartum period were not included. Demographic, clinical, hemodynamic and echocardiographic data as well as data on serology were evaluated. Data on endomyocardial biopsies and coronary angiography were reviewed. The outcomes of the different treatment strategies as well as their complications were evaluated.

## Results

Over a 10-year period six PPCM patients were treated with mechanical support for acute heart failure at our center (Table 1). All six patients were treated with an IABP and one patient was treated with ECMO. Four patients were implanted with a continuous-flow LVAD (HeartMate II^®^, Thoratec Inc., Pleasanton, California, USA), three of them were transplanted and one patient is still on the transplant waiting list. The mean age at presentation was 34.7 years, the mean body surface area (BSA) was 1,76 m^2^. Five patients were Caucasian, one was native African. All patients but one were multiparous with the number of pregnancies ranging from two to four. Serology was examined for Coxsackie virus B1-5, Mycoplasma pneumoniae, toxoplasmosis, hepatitis B and C, HIV, Ebstein-Barr and adeno- and entero-virus in all patients. Active infection with Mycoplasma pneumoniae was found in two patients but active myocarditis was excluded by means of endomyocardial biopsy. Endomyocardial biopsies in two other patients, taken at the time of placement of the LVAD, were also negative for myocarditis.

**Table 1:** Patient characteristics

Patient	1	2	3	4	5	6
**Year**	2001	2008	2010	2007	2008	2009
**Age (y)**	34	35	36	37	38	28
**BSA (m^2^)**	1.90	1.88	1.58	1.83	1.60	1.79
**Race**	C	A	C	C	C	C
**Obstetrical Hx**	G4A0P4	G2A0P2	G4A0P4	G1A0P0	G2A0P1	G3A1P1
**Symptom onset**	3wPP	5mPP	5mPP	38wPr	35wPr	38wPr
**Diagnosis**	3w PP	10mPP	18mPP	38wPr	36wPr	38wPr
**Clinical picture**	APE	ADHF	ADHF	ADHF	ADHF	CS
**LVEDD (mm)**	55	62	79	53	61	68
**Serology**	Negative	Mycoplasma IgM	Negative	Negative	Negative	Myocplasma IgM
**Coronary angio**	Normal	-	Normal	-	Normal	-
**Biopsy**	-	Negative	Negative	-	Negative	Negative
**IABP (d)**	7	13	5	4	6	1
**ECMO (d)**	-	-	-	-	-	7
**LVAD (d)**	-	126	Since 26/04/2010	-	360	78
**Complications**	-	Perop. rupture aorta Tamponade 2x Pocket Infection	Rectus hematoma Occlusion AFC	-	-	Bleeding anast. aorta
**Outcome**	Recovery	Tx, SD 535 days postTx	Alive, on Tx list	Recovery	Tx	Tx

A, African; APE, acute pulmonary edema; ADHF, acute decompensated heart failure; AFC, Arteria Femoralis Communis; anast, anastomosis; BSA, body surface area; C, Caucasian; CS, cardiogenic shock; d, days; Hx, history; LVEDD, left ventricular end diastolic diameter at presentation; Perop, peroperative; PP, postpartum; Pr, pregnancy; SD, sudden death; Tx, cardiac transplantation; Y, year.

### Presentation in the postpartum period

Patient 1 was a 34-year-old patient (G4A0P4) who presented with acute pulmonary edema 16 days after delivery of a healthy son. She was initially treated with intravenous diuretics and vasodilators, but her condition only stabilized after insertion of an IABP. After initiation of conventional heart failure therapy with ACE-inhibitors, diuretics and low dose beta-blockers the patient was easily weaned off the IABP and discharged home four weeks after admission. She is still in follow-up and doing well under treatment with beta-blocking agents.

Patient 2 was a 35-year-old South African woman (G2A0P2) who developed progressive dyspnea from the fifth month postpartum. She came to the Emergency Room a few months later with a clinical picture of severe decompensated heart failure with lactate acidosis and liver failure. After initiation of inotropic therapy and IABP insertion her condition stabilized with complete resolution of the lactate acidosis and liver function. Despite initiation of proper heart failure therapy, weaning off the IABP was not possible and the implantation of a LVAD was decided. The implantation was complicated by a rupture and large hematoma of the descending aorta for which an endoprosthesis was inserted. During the early postoperative phase 2 revisions were necessary because of pericardial tamponade. Long-term antibiotic therapy was initiated because of infection of the pocket. After a long postoperative period the patient could be mobilized and discharged home 67 days after placement of the LVAD. No recovery in left ventricular function was noted during follow-up. A total of 126 days after implantation of the LVAD she was successfully transplanted and did well. Unfortunately she died suddenly two years later, she developed electromechanical dissociation during hospitalization for heart failure due to mild rejection, prolonged resuscitation was unsuccessful. An autopsy was not performed.

Patient 3, a 36-year-old mother of four children, presented very late in the postpartum period (18 months postpartum), she developed progressive symptoms of heart failure during the first months after her last delivery. She presented with cachexia and decompensated heart failure. The left ventricular end-diastolic diameter was 79 mm at presentation. After minor decongestion with diuretics, low dose dopamine was started and an IABP was inserted because of refractory hypotension and low output failure. Five days later an LVAD was implanted electively because of lack of left ventricular recovery and the impossibility to wean the patient off the IABP and dopamine. The postoperative course was complicated by a spontaneous rectus hematoma at the 11^th ^postoperative day (supratherapeutic prothrombin time) and a thrombotic occlusion of the right common femoral artery. The arterial occlusion was a consequence of the IABP and a thrombectomy was performed at Day 35 post LVAD with good clinical resolution afterwards. During ambulatory follow-up, left ventricular end diastolic diameter decreased from 79 to 72 mm without recovery of left ventricular function.

### Presentation late in pregnancy

Two patients presented with acute decompensated heart failure and were in New York Heart Association class III. An IABP was inserted in both patients prior to caesarian section.

Patient 4, a 37-year-old nullipara could be weaned off the IABP four days later and is still under treatment with conventional heart failure therapy and is doing well.

Patient 5, a 38-year-old woman (G2A0P1) could be weaned off the IABP after six days but remained symptomatic the following weeks with severe hypotension necessitating a continuous dopamine infusion. She was treated with bromocriptine but remained inotrope-dependent. She was implanted with a LVAD 21 days after removal of the IABP. There were no complications. Follow-up echocardiography showed some recovery of left ventricular function but the right ventricular function remained moderate; a trial to remove the LVAD was not attempted. She was successfully transplanted almost one year after LVAD placement and is still doing well.

The sixth patient, a 28-year-old G3A1P1 developed rapidly progressive dyspnea at the end of pregnancy. Heart failure was initially not recognized and delivery was induced with prostaglandins. Afterward she rapidly progressed to cardiogenic shock. She was referred to our center. During transport a continuous infusion with adrenaline was initiated because of severe shock. Upon arrival the patient was immediately intubated, meanwhile an IABP was percutaneously inserted. A stillborn baby was delivered by caesarean section. The patient remained in shock with severe lactate acidosis and multiple organ dysfunction syndrome despite treatment with dobutamine, levosimendan and high doses of noradrenaline. Her condition worsened rapidly, she was not stable enough for implantation of a LVAD. An ECMO was percutaneously inserted at the bedside without complications. The system comprised a Medos Hilite 7000 LT oxygenator (Medos Medizintechnik AG, Stolberg, Germany) and a Sorin revolution centrifugal pump (Sorin Group, Arvado, Colorado, USA) (18 Fr arterial line: femoral approach, 18 Fr venous line: jugular approach). The following days we noted respiratory and metabolic improvement. Because of the absence of left ventricular recovery a LVAD was implanted after seven days of ECMO. There was a revision at Day 1 because of bleeding at the anastomosis of the aortic cannula. During the postoperative course she was treated for ventilator associated pneumonia with complete recovery. Sildenafil treatment for moderate right ventricular function and pulmonary hypertension was initiated at the fourth day post-LVAD implantation until transplant. She was discharged home 37 days after initial admission and was successfully transplanted 78 days after LVAD implant and is doing well up till now.

## Discussion

We describe six well-documented cases of severe PPCM that presented with acute heart failure requiring mechanical support. The diagnosis was based upon development of symptoms of heart failure in the last month of pregnancy or during the first five months after delivery without arguments for pre-existing structural heart disease. In each patient an extensive work-up was performed to exclude other causes of heart failure. Two patients had arguments for active Mycoplasma pneumoniae infection, but myocarditis was excluded by means of endomyocardial biopsies.

We describe short- and/or long-term mechanical support when intensive medical therapy fails to stabilize a PPCM patient with severe heart failure. Mechanical short-term support can be provided percutaneous with IABP or ECMO. An IABP can easily be placed at the bedside and has little side effects in this young patient population. There are no randomized data on the use of IABP in non-ischemic refractory heart failure and European guidelines recommend insertion of an IABP when inotropes fail to restore the blood pressure and signs of hypoperfusion persist [27]. In our series the use of IABP up to 13 days was complicated by one thrombotic occlusion of the common right femoral artery, which was corrected uneventfully after thrombectomy. All patients treated with IABP were anticoagulated with unfractionated heparin (UFH) aiming at an activated partial thromboplastin time (aPTT) of 65 to 85 seconds. Weaning from the IABP is usually attempted over one to three days by gradually decreasing the 1:1 support to a 1:2 and a 1:3 support. If a 1:3 support is well tolerated for at least four hours, the IABP is removed. When weaning off the IABP is not possible, the IABP is removed at the time of implantation of the ECMO or the LVAD.

There is a current trend to use short-term support with ECMO in refractory cardiogenic shock but data from large randomized trials are lacking. ECMO is considered an emergency rescue therapy for patients with refractory cardiogenic shock; their condition is so unstable that they are not eligible for immediate LVAD implantation. The ECMO can be inserted at the bedside; it is relatively cheap (as compared to the implantable LVAD) and gives the treating physicians some time to wait for recovery or a more stable condition. However, close monitoring of the coagulation parameters is needed and it is, therefore, labour-intensive. Patients on ECMO are treated with UFH in order to obtain an activated clotting time (ACT) of 170 to 200 seconds. Antithrombin III (ATIII) levels are analyzed daily, ATIII concentrate is given if the ATIII activity drops below 70%. A visco-elastic coagulation measurement is checked twice daily or whenever bleeding occurs. Patients on arterio-venous ECMO are ventilated with conventional settings, with a FiO_2 _to achieve an acceptable PaO_2 _(at least 60 mmHG). Inotropic support is reduced and stopped but milrinone is often continued for its dilator properties and positive effects on microcirculation. Fluid management is aimed at preserving renal function and ensuring a stable circulation. As ECMO flow depends on right atrial filling, this is monitored by means of echocardiography. During a weaning attempt each partial decrease in ECMO flow should be compensated by an increase in stroke volume without excessive increase in inotropic support. There are three case reports on ECMO in PPCM where ECMO served as a safe bridge to recovery [14-16]. In our series ECMO was used in one patient because of refractory cardiogenic shock and multiple organ dysfunction syndrome one-day post caesarean section. ECMO allowed hemodynamic and metabolic stabilization. In contrast to the above mentioned case reports we saw no recovery of left ventricular function and in our patient ECMO served as a bridge to LVAD.

LVADs offer a more long-term support. In a recent position statement Sliwa *et al*. promote the use of a mechanical assist device in PPCM in case of refractory heart failure despite optimal medical therapy [28]. The continuous-flow HeartMate II was introduced in 2004 and has shown improvement in survival, reduction in adverse events and improved functional capacity [25]. This axial flow pump draws the blood on a continuous basis from the left ventricle via an apical drainage cannula and propels it back into the aorta by a rotary pump in a nonphasic flow pattern. Its smaller size makes it suitable for patients with a low BSA, which is frequently the case in this young female population. After implantation of the LVAD NO-ventilation is routinely applied in our center to support the right ventricle, inotropic support is gradually decreased and replaced by oral heart failure therapy. Echocardiography is used to assess left ventricular filling, a neutral interventricular septum position indicates adequate left ventricular filling. Bleeding complications in the immediate postoperative phase still pose a problem but recent data on the HeartMate II device support a less aggressive anticoagulation protocol [18,22]. More recently late bleeding complications up to 44.3% have been observed in continuous-flow LVAD patients, possibly due to an acquired von Willebrand Syndrome [29]. In our center the antithrombotic regimen is started as soon as drain output reaches levels of 50 ml/h or less. It comprises Aspirin 100 mg and Enoxaparin 40 mg once daily (20 mg in case of GFR < 30 ml/minte, 60 mg in case of body weight >90 kg). Acenocoumarol (target INR 1.5 to 2) is started as soon as a more stable hemodynamic condition is reached and in the absence of bleeding. Bleeding complications were observed in three patients during the early postoperative phase, with need for revision in two patients. We observed no late bleeding complications. Infection of the pocket with the need for long-term antibiotic treatment occurred in one patient. There were no thrombotic complications related to the LVAD, despite the fact that PPCM is a pro-thrombotic condition. Right heart failure, defined as the postoperative need for temporary right ventricular mechanical or inotropic support for more than 14 days following implantation, was not noted although one patient was treated until transplantation with a low dose Sildenafil. Neurological complications did not occur in this small series. Sufficient recovery of left ventricular function to allow LVAD explantation is rare but has been described in PPCM patients treated with pulsatile devices, we found no data on explantation of continuous-flow devices in PPCM patients. In our series we saw a decrease in left ventricular end-diastolic diameter and some improvement in left ventricular function, but the right ventricular function remained moderate. In our opinion the decrease in left ventricular end diastolic diameter (LVEDD) and improvement in left ventricular function can be attributed to the unloading of the left ventricle.

These six patients presented over a wide time range between 2001 and 2010 with a trend towards an increasing incidence over the last three years at our center, this stresses the need for a national and international registry for this pathology. One could argue that the therapy has become more invasive over the years; the first two patients being managed with IABP alone. However, the more invasive therapy (ECMO, LVAD) in the patients who presented later is attributable to the more severe condition of these patients with the inability to wean the patients off IABP and/or intravenous inotropes.

Despite the fact that bromocriptine appears promising as a novel disease-specific treatment, we initiated it briefly in one patient (patient 5) and stopped it after implantation of the LVAD. Currently it is not clear whether the results of the proof of concept study by Sliwa *et al*., where bromocriptine was added to standard heart failure therapy (ACE-inhibitors, aldactone, betablockers and diuretics), can be extrapolated to this patient population dependent of IV inotropes and/or mechanical support. We hope that future trials will address this question.

## Conclusions

**Figure 1:**
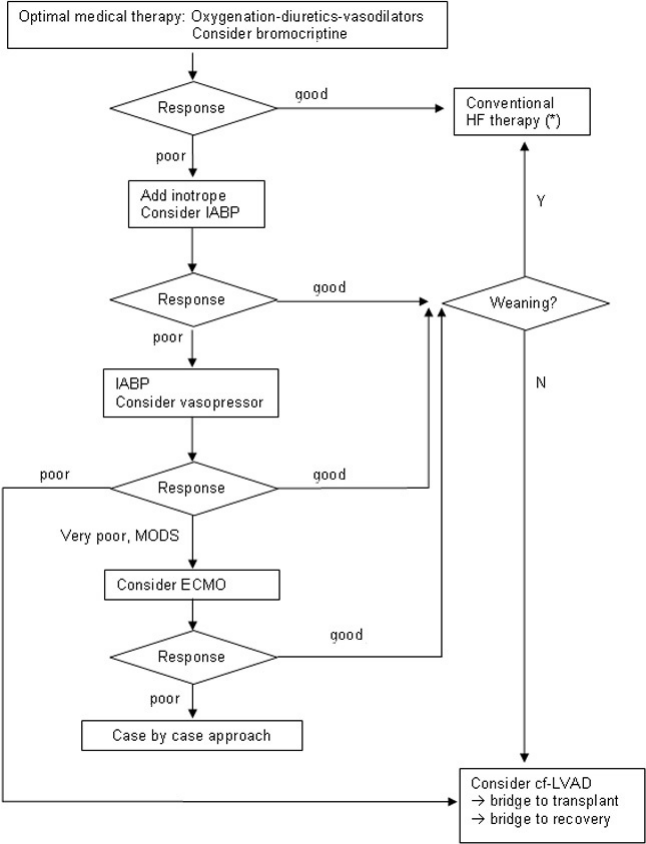
**Algorithm for the treatment of acute and critically ill PPCM**. PPCM, peripartum cardiomyopathy; HF, Heart Failure; (*) = Avoid ACE-inhibitors, angiotensin II receptor blockers and aldosterone antagonists during pregnancy; cf-LVAD, continuous-flow left ventricular device; ECMO, extracorporeal membrane oxygenation; IABP, intra aortic balloon pump; MODS, multiple organ dysfunction syndrome.

In acute and critically ill PPCM patient's, mechanical support with IABP, even prior to delivery, is safe and feasible and serves as a bridge to partial recovery or as a bridge to LVAD. ECMO can serve as a bridge to LVAD, in case of refractory cardiogenic shock despite IABP and full inotropic support. The newer continuous-flow assist devices are a safe bridge to transplant for PPCM patients who cannot be weaned off intravenous inotropic support or mechanical support with IABP or ECMO. The role of bromocriptine treatment in these patients needs to be explored in future trials. Based on the literature and our experience we propose an algorithm for the treatment of acute and critically PPCM (Figure 1).

## Key messages

• Acute and critically ill PPCM patients, refractory to medical therapy, should be treated with mechanical support.

• IABP support is feasible and safe in the pre- and postpartum period as a bridge to recovery or as a bridge to assist device.

• In patients with refractory cardiogenic shock and Multiple Organ Dysfunction Syndrome despite IABP, ECMO should be considered as a temporary 'emergency rescue' support (bridge to recovery or bridge to LVAD).

• Continuous-flow left ventricular assist devices are safe as a bridge to transplant in this young patient population.

• Bromocriptine, a novel disease-specific treatment, can be considered in these patients.

## Abbreviations

ACT: activated clotting time; aPTT: activated partial thromboplastin time; ATIII: antithrombin III; ECMO: extra corporeal membrane oxygenation; IABP: intra aortic balloon pump; LVAD: left ventricular assist device; LVEDD: left ventricular end diastolic diameter; PPCM: peripartum cardiomyopathy; UFH: unfractionated heparin.

## Competing interests

The authors declare that they have no competing interests.

## Authors' contributions

The idea for the article came from SG and MDP. SG, MDP, EVDC, FT, FM and IH collected data and prepared the article. YVB, SB and FDS critically reviewed the paper. SG and MDP finalized the text. All authors read and approved the manuscript.
